# Substantially enhanced plasticity of bulk metallic glasses by densifying local atomic packing

**DOI:** 10.1038/s41467-021-26858-9

**Published:** 2021-11-12

**Authors:** Yuan Wu, Di Cao, Yilin Yao, Guosheng Zhang, Jinyue Wang, Leqing Liu, Fengshou Li, Huiyang Fan, Xiongjun Liu, Hui Wang, Xianzhen Wang, Huihui Zhu, Suihe Jiang, Paraskevas Kontis, Dierk Raabe, Baptiste Gault, Zhaoping Lu

**Affiliations:** 1 Beijing advanced innovation center for materials genome engineering, State Key Laboratory for Advanced Metals and Materials, University of Science and Technology Beijing, 100083 Beijing, China; 2 Institute for Advanced Materials and Technology, University of Science and Technology Beijing, 100083 Beijing, China; 3 Max-Planck-Institut für Eisenforschung GmbH, Department of Microstructure Physics and Alloy Design, Max-Planck-Strasse 1, 40237 Düsseldorf, Germany; 4 Department of Materials, Imperial College London, Kensington, London, SW7 2AZ UK

**Keywords:** Metals and alloys, Mechanical properties

## Abstract

Introducing regions of looser atomic packing in bulk metallic glasses (BMGs) was reported to facilitate plastic deformation, rendering BMGs more ductile at room temperature. Here, we present a different alloy design approach, namely, doping the nonmetallic elements to form densely packed motifs. The enhanced structural fluctuations in Ti-, Zr- and Cu-based BMG systems leads to improved strength and renders these solutes’ atomic neighborhoods more prone to plastic deformation at an increased critical stress. As a result, we simultaneously increased the compressive plasticity (from ∼8% to unfractured), strength (from ∼1725 to 1925 MPa) and toughness (from 87 ± 10 to 165 ± 15 MPa√m), as exemplarily demonstrated for the Zr_20_Cu_20_Hf_20_Ti_20_Ni_20_ BMG. Our study advances the understanding of the atomic-scale origin of structure-property relationships in amorphous solids and provides a new strategy for ductilizing BMG without sacrificing strength.

## Abstract (web summary)

Common wisdom to improve ductility of bulk metallic glasses (BMGs) is to introduce local loose packing regions at the expense of strength. Here the authors enhance structural fluctuations of BMGs by introducing dense local packing regions, resulting in simultaneous increase of ductility and strength.

## Introduction

Bulk metallic glasses (BMGs) inherit a disordered amorphous structure from the liquid state^1^. Due to the lack of crystalline defects which serve as low-barrier deformation carriers, such as dislocations and stacking faults, BMGs are generally much stronger and harder than their crystalline counterparts^2,3^. However, BMGs usually fail catastrophically under load with negligible tensile plasticity at room temperature^4^, which seriously hinders their widespread use. Unlike their crystalline counterparts whose properties are tunable via microstructural engineering^5^, the disordered atomic packing of BMGs is not readily accessible to a quantitative description and only limited methods exist for tuning their structure-property relationships^6–8^. Tailoring the mechanical properties of BMGs to overcome their room-temperature brittleness has hence remained a long-standing challenge.

Plastic deformation in BMGs well below their glass transition temperature mainly arises from local diffusive jumps^8^, or some local events of cooperative shearing of atomic clusters termed shear transformation zones (STZs)^9,10^, whereby a group of atoms cooperatively overcomes the saddle point of the energy barrier for local atomic rearrangement^11,12^. The deformability originates from the flexibility inherent to the metallic bonding: the delocalized electrons allow metal atoms to slide past one another without being subjected to bond breaking that would favor damage over shear for instance in ionic glasses^11^. Although the site where local shear transformation starts is still hard to predict, it is well accepted that introducing more loosely packed regions is effective in facilitating local plastic events in BMGs^13,14^. These regions are associated with a high local potential energy and prone to inelastic deformation upon loading, exhibiting a liquid-like behavior^13^. As a consequence, enhancing the number of loosely packed regions was found to be effective in improving the plasticity of BMGs. This material design route led to improvement in plasticity of BMGs through approaches such as cryogenic thermal cycling^14^ or severe plastic deformation^15^, which usually enhance structure fluctuations via an increased availability of less dense regions. However, most current approaches for improving plasticity of BMGs usually reduce the thermal stability and yield strength due to the introduction of more loosely packed regions^16^. In contrast, annihilation of loosely packed regions is usually believed to enhance strength and hardness, and improve thermal stability, but tends to deteriorate plasticity, as demonstrated by the annealing-induced embrittlement in BMGs^17^.

Here, we report on a novel design concept for improving deformability of BMGs. We increase the structural fluctuation of BMGs via doping with nonmetallic elements (NMEs) which have a small atomic size and a large negative heat of mixing with the elements constituting the BMG. Candidate elements that we selected are oxygen, nitrogen, carbon and boron, each added individually to Ti-, Zr-, and Cu-based BMGs. We identify particularly suited doping regimes (ranging from 0.1% to 0.3%) where we observed an appreciable rise in strength and ductility. This can be ascribed to the increase in the volume fraction of local dense packing regions (LDPRs) forming around these nonmetallic solutes, whilst avoiding the formation of brittle secondary phases. The neighborhood of these LDPRs becomes relatively more loosely packed, which consequently enhances the material’s structural fluctuation, promotes localized shearing and dramatically increases the macroscopic plasticity and toughness, along with on enhancement in strength. Guided by thermodynamics, in terms of an adequate negative heat of mixing associated with these dopants, our approach is in principle universal and could be deployed to improve the properties of a wide range of MGs.

## Results

### Mechanical properties of BMGs after doping

**Fig. 1:**
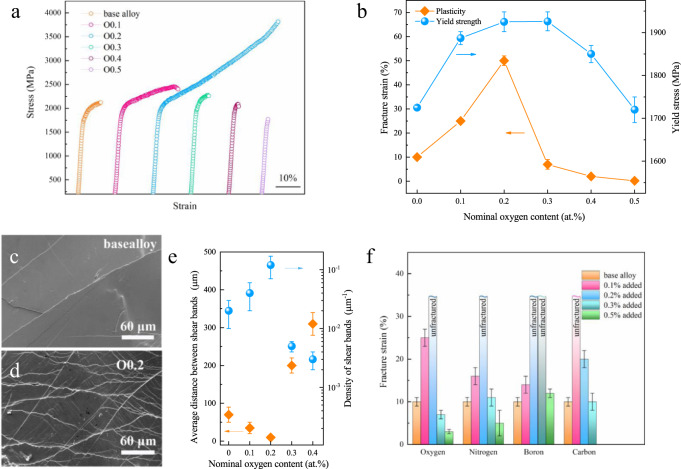
Mechanical behavior of the base and doped ZrTiHfCuNi BMGs. Compressive stress-strain curves of ZrTiHfCuNi BMGs doped with a different content of oxygen (**a**). Variation of fracture strength and plasticity with addition of oxygen (**b**). Lateral surface of fractured base alloy (**c**) and O0.2 (**d**). Shear band spacing and number density in the fractured samples with a different content of oxygen (**e**). Plasticity change with doping in ZrTiHfCuNi BMGs (**f**). The error bars in **b**, **e**, **f** were estimated from standard deviation with a confidence of 95%.

First, we applied this design concept by doping of 0.1–0.3 at.% oxygen into a series of typical Ti-, Zr-, and Cu-based BMG systems. Enhanced plasticity and strength were observed in these alloy variants (Supplementary Fig. 1a–c). We also studied the equiatomic Zr_20_Cu_20_Hf_20_Ti_20_Ni_20_ BMG doped with 0.1, 0.2, 0.3, and 0.5 at.% of one of the NMEs oxygen, nitrogen, carbon, and boron. We then focused on the BMG doped with oxygen (Zr_20_Cu_20_Hf_20_Ti_20_Ni_20_)_100−*x*_O_*x*_ (*x* = 0.1, 0.2, 0.3, 0.4, and 0.5 at.%), which will be referred to as O0.1, O0.2, O0.3, O0.4, and O0.5, respectively. X-ray diffraction confirms that the samples are amorphous (Supplementary Fig. 2). Figure 1a shows the compressive stress-strain curves at room temperature for the base and oxygen-containing BMGs. The average yield strength σ_y_ rises from 1725 ± 25 MPa for the base alloy to 1887 ± 15 MPa and 1925 ± 23 MPa for the samples with 0.1 and 0.2 at.% oxygen, respectively. More strikingly, the fracture strain was significantly increased from ~8% for the base alloy up to >25% (i.e., unfractured under compression) for the O0.1 and O0.2 BMGs, respectively. Further increasing the oxygen content decreases the fracture strain. The sample with 0.5 at.% oxygen (i.e., O0.5) fractures immediately after yielding without any plasticity.

Doping with oxygen strengthens and improves plasticity (Fig. 1b). This enhanced plasticity is reflected by the proliferation of shear bands (Fig. 1c–e). Scanning electron micrographs show that the shear band spacing in alloy O0.2 was reduced to ~5 µm from ~70 µm in the base alloy, whilst the shear band number density increased from ~0.02 µm^−1^ in the base alloy to ~0.1 µm^−1^ accordingly, implying that more local regions can be activated to create local plastic events after an optimal oxygen addition. Doping the base BMG with 0.1–0.2% of boron, carbon, or nitrogen also substantially improved plasticity, as summarized in Fig. 1f (see Supplementary Fig. 3 for the corresponding stress-strain curves). More interestingly, doping of the reference alloy with 0.1–0.2 at.% NMEs also leads to a remarkable increase in both tensile ductility and fracture toughness (Supplementary Figs. 4 and 5). When exposed to tensile loading, the base alloy and the BMGs doped excessively with these small atoms, e.g., alloy O0.4, fractured catastrophically with no appreciable ductility. However, a remarkable total plastic strain of 0.2–0.5% was recorded for the medium-doped O0.2 alloy (Supplementary Fig. 4). In addition, the fracture toughness is also significantly increased from 87 ± 10 MPa√m for the base alloy to 165 ± 15 MPa√m for the O0.2 alloy, and then decreased to 30 ± 5 MPa√m for the over-doped O0.4 alloy (Supplementary Fig. 5).

### Structural change of BMGs after doping

**Fig. 2:**
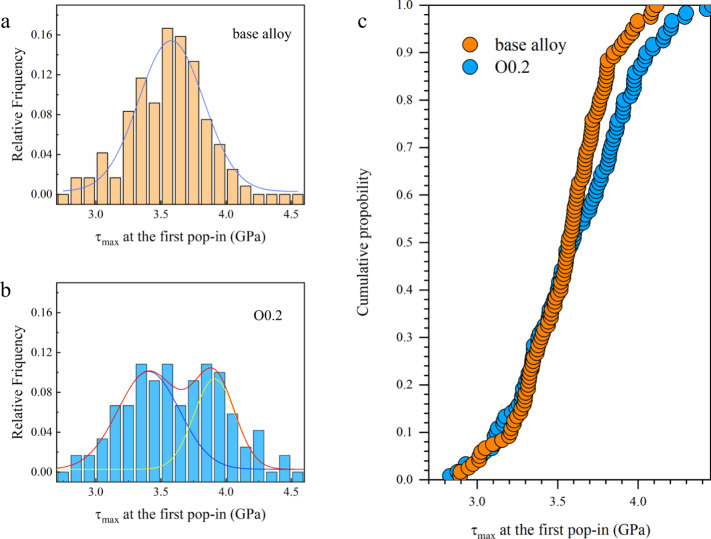
Relative frequency distributions of *τ*_max_ probed by nanoindentation of the base alloy. **a** and alloy O0.2 (**b**), and the corresponding cumulative probability of *τ*_max_ (**c**). Relative frequency distributions of *τ*_max_ of O0.2 in **b** shows a bimodal characteristic and can be fitted by two Gaussian curves.

Ductilization of monolithic BMGs is generally attributed to the increase in the structural heterogeneity from the availability of local loosely packed regions (LLPRs), thus usually leading to a decrease in the critical shear stress for local plasticity^13,14,18,19^. The variation of the critical shear stress values required for triggering local plastic deformation events in O-doped BMGs was then checked (Fig. 2). In nanoindentation of BMGs, the initial local yielding is indicated by a sharp ‘pop-in’ (abrupt increase in indentation depth *h*), visible on the load-*h* curve (as shown in Supplementary Fig. 6), corresponding to the transition of purely elastic to elasto-plastic deformation. The relative frequency distribution *vs*. the maximum shear stress (*τ*_max_) at the first pop-in for the base BMG and the O0.2 variant is shown in Fig. 2a, b, respectively, whilst the corresponding cumulative probability plot is shown in Fig. 2c. It can be seen that the low-stress portion of the distribution curves remains almost unchanged after doping with oxygen. However, the high-stress portion of the distribution curves shifts towards larger values, which is related to the higher critical shear stresses required to trigger local shearing events in alloy O0.2, suggesting that the reason for the plasticity enhancement in the O-doped BMG cannot be attributed to the increase of LLPRs.

It has been proposed that, to some degree, the elastic fluctuations from nanoindentation can be ascribed to structural fluctuation at different length scales^20^. The relative width of the *τ*_max_ distribution was thus regarded as a reflection of the structural fluctuation of the material^17,21,22^. Clearly, the relative frequency distribution in the case of O0.2 BMG shows a bimodal characteristic and can be fitted by two Gaussian functions (see Fig. 2b). The distribution width of the O0.2 BMG is substantially wider compared to the base BMG, mainly due to a shift and a spread of the pop-in events toward a higher stress level. The wider distribution of *τ*_max_ in O0.2 manifests enhanced structural fluctuations but is not a result of the increment in LLPRs. Rather, this bimodal characteristic reveals that the formation of local “stiffened” regions via oxygen addition is feasible for promoting structural heterogeneity.

**Fig. 3:**
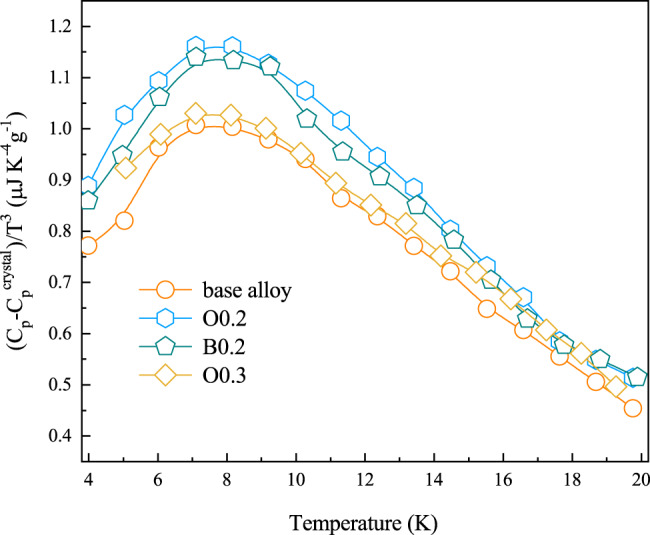
Experimental data of low-temperature specific heat capacity of the base alloy, and of the doped alloys O0.2, B0.2, and O0.3. (*C*_p_−*C*_p_^crystal^)/*T*^3^ vs *T*, in which the heat capacity of the crystallized counterpart (*C*_p_^crystal^) of the as-cast amorphous sample was subtracted from that of the glassy sample (*C*_p_). Alloys O0.2 and B0.2 show higher boson peak intensity, as compared with the base alloy, indicating a state of enhanced structural heterogeneity.

For BMGs lacking long-range order, their vibrational density of state usually display an excess peak over that of their crystalline counterparts which can be approximated using the Debye square-frequency law at low frequencies. This excess contribution to the vibrational spectrum is referred to as boson peak, and it originates from the intrinsic structural heterogeneity of glasses^15,23–25^. Figure 3 evidences a boson peak^24^ almost at the same temperature of ~7 K for all four tested BMGs. The peak intensity ${H}_{{{{\rm{BP}}}}}$ for the O0.2 and B0.2 is ~20% higher than in the base BMG at ~1.2 μJ K^−4^g^−1^ and 1.0 μJ K^−4^g^−1^, respectively, indicating a more heterogeneous atomic packing after nonmetallic doping.

Furthermore, Vickers hardness and density were found to increase with doping of oxygen and approach a maximum for the O0.2 BMG which shows a global densification after doping with oxygen (Supplementary Fig. 7). Similar trends were also found for doping of other NMEs as shown in Supplementary Table 1. It has been proposed before that the molar volume of BMGs follows a negative relationship with yield strength^26^. The increased density translates into a lower molar volume and thus a higher yield strength. In addition, the glass-forming ability (GFA) of alloys doped with 0.1, 0.2, and 0.3 at.% oxygen is enhanced as compared to the base BMG (Supplementary Fig. 8). It is well accepted that the GFA is positively correlated with density^4,27,28^. The increased GFA thus provides another hint toward higher density in the O-doped alloys.

The actual content of oxygen in all the alloys investigated was quantified by atom probe tomography (APT) and inert gas fusion (IGF) probing. As shown in Supplementary Table 2, the measured oxygen content is 0.05–0.09 at.% higher than the target value, but the alloys with a high nominal content also show consistently a high measured value, confirming that the experimental method for adding oxygen is well controlled. The three-dimensional elemental distribution of all these alloys was further investigated by APT. The metallic elements were found to have a uniform distribution on the scale probed by APT, both in the base alloy and in the alloy variant O0.2 (Supplementary Fig. 9). Neither oxides nor secondary phases were found by APT and transmission electron microscopy (Supplementary Fig. 10) so that the increase in the hardness and density cannot be ascribed to precipitates. With further addition of oxygen, significant segregation of this element was found. Supplementary Figure 9 shows a 30-nm-wide O-segregated region in alloy O0.5, indicating that excessive oxygen doping can lead to the formation of brittle oxides, which explains the observed deterioration of the alloy’s plasticity.

**Fig. 4:**
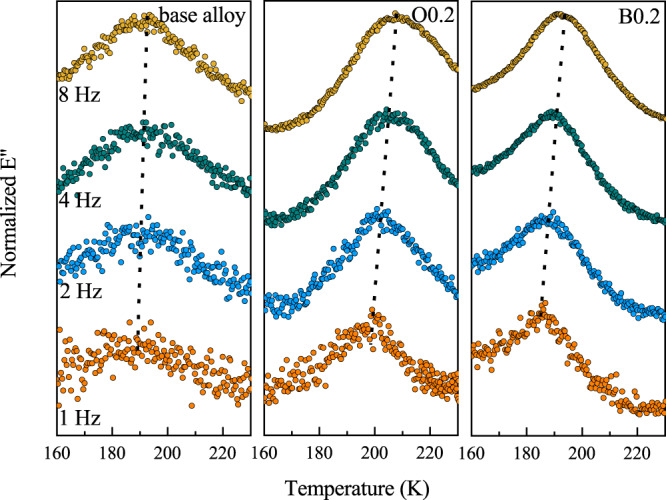
The *γ* relaxation in BMGs at cryogenic temperatures. Comparison of isochronal relaxation spectrum for the base alloy, and for the doped alloys O0.2 and B0.2 within the range of 160–230 K at discrete frequencies of 1 Hz, 2 Hz, 4 Hz, and 8 Hz. A more pronounced *γ* relaxation with lower activation energy was observed in the alloys O0.2 and B0.2.

The question about whether the increased structural heterogeneity from enhancing the availability of local densely packed regions can facilitate local atomic reshuffling was then naturally raised. A local relaxation process, which involves the most mobile or anelastically moving atoms, referred to as fast-secondary or γ relaxation, was recently discovered to be the precursory process of local shearing^29^. We performed isochronal dynamic mechanical analysis (DMA) measurements to reveal the local relaxation characteristics in O-doped BMGs. Figure 4 illustrates the temperature dependence of the normalized loss modulus, *E″*, for the base alloy, and for the doped alloys O0.2 and B0.2 at cryogenic temperatures for frequencies *f* of 1, 2, 4, and 8 Hz. The γ relaxation process^29^ with a distinctive and complete *E*″ peak for the BMGs can be clearly seen in the temperature range of 160–210 K at these frequency values. As the frequency increases from 1 to 8 Hz, the *E*″(*T*) curve gradually shifts towards a higher temperature. The activation energy *E*_γ_ for O0.2 alloy was estimated to be 0.57 ± 0.07 eV (see Methods section and Supplementary Table 3), i.e. lower than in the base alloy (i.e., 0.69 ± 0.08 eV) and in the O0.3 alloy (i.e., 0.60 ± 0.07 eV). With addition of a small amount (0.1−0.3%) of other NMEs such as B, C, or N, *E*_γ_ also decreased significantly to ~0.57–0.60 eV (Supplementary Figs. 11 and 12). Note that the *E*_γ_ value is related to the energy barrier for enabling rearrangement of local anelastic clusters. In addition, the intensity (*H*) and temperature span (*W*) increase from 125 MPa and 60 K for the base alloy to 190 MPa and 65 K for the alloy O0.2, respectively (Supplementary Fig. 11). This indicates a larger volume fraction of the so-called fertile regions^30,31^ which can be more easily activated during the *γ* relaxation process in the alloy O0.2. With further increase in the oxygen content, however, the value of *H* decreases to 140 MPa in the alloy O0.3, indicating a decreased *γ* relaxation process. Overall, the decrease in activation energy for the γ relaxation indicates that the rearrangement of local anelastic clusters is greatly facilitated by our doping strategy.

### Structural evolution of the doped BMGs during plastic deformation

**Fig. 5:**
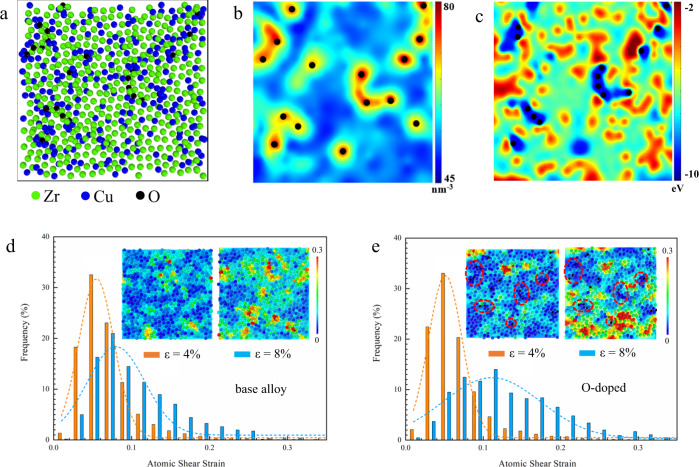
MD simulations of local atomic packing and shear response of the base alloy and alloy with O doped. A slice (6.25 × 6.25x × 0.35 nm^3^) taken from a cubic box with 13,500 atoms is shown as a representative example of atomic packing in O-doped MGs. **a** shows that local regions around doped O atoms are more densely packed. Atomic number density (**b**) and potential energy (**c**) mapping of the O-doped alloy. The distribution histogram of local shear strain for the base (**d**) and O-doped alloy (**e**) at strains of 4% and 8%. Insets in **d** and **e** are the atomic shear strain map at a strain of 4% and 8%.

To rationalize the structural influence of doping, molecular dynamics (MD) simulations were conducted in a simplified Zr_60_Cu_40_ metallic glass with different content of oxygen. Based on the relative enthalpy of formation of their oxides, we assumed that the behavior of Ti and Hf atoms is relatively similar to that of Zr, and Ni similar to Cu. The detailed calculation method can be found in the Supplementary Materials. A 6.25 × 6.25 × 0.35 nm^3^ slice cut from a cubic box with 13,500 atoms (Supplementary Fig. 13) was taken as a representative volume of the O-doped BMG (Fig. 5a). The atomic number density was quantitatively evaluated based on Voronoi tessellation^32^. For the Zr_60_Cu_40_ alloy mimicking the base alloy, the average density ranges from 47 nm^−3^ to 59 nm^−3^, with an average of 53 nm^−3^ (Supplementary Fig. 13) which is consistent with the value calculated from the molar volume (54 nm^−3^)^33^. Doping oxygen increases the average density to 55 nm^−3^ with a much wider range from 46 nm^−3^ to 82 nm^−3^. In the vicinity of O atoms, the average density ranges from 65 nm^−3^ to 82 nm^−3^, forming local dense packing regions (LDPRs), surrounded by regions of much lower density ranging from 46 nm^−3^ to 65 nm^−3^ (Fig. 5b). The packing efficiency was also calculated as the volume ratio of the atoms within one unit cell to the unit cell itself^34,35^. The average packing efficiency of the O-centered clusters was determined to be 0.925, which is much higher than that of the icosahedron clusters (0.707) and the average packing efficiency of the sample (0.703), indicating a much higher packing efficiency of the O-centered regions. Figure 5c shows the potential energy maps of the alloy with O addition. The global potential energy of the O-containing system decreases as a result of O addition, indicating a more stable glassy structure, as expected from the negative enthalpy of mixing. The potential energy of Zr atoms near O ranges from −6.8 eV to −10.3 eV, which gives rise to a much wider distribution of potential energy of Zr (from −5.8 to −10.3 eV) in the O-containing alloy, as compared with that in the base Zr_60_Cu_40_ alloy (from −5.5 to −6.7 eV, Supplementary Fig. 14). Similarly, the addition of O also substantially changed the potential energy distribution of Cu atoms, as demonstrated in Supplementary Fig. 14. Doping with O hence decreases the potential energy of the system, conversely to traditional approaches and increases the average atomic density. The interactions of O with Zr and Cu forms O-centered LDPRs (O-LDPRs) that triggers density fluctuations, thereby enhancing structural heterogeneity and altering the mechanical response.

The origin of the increased packing density was then checked based on the O-metal bonding status. Generally, the covalency degree of the O-metal bonding can be revealed by an electron localization function (ELF)^36^. For ELF = 1, the bonding is covalent with perfect electron localization and for ELF = ½, the bonding is metallic, i.e., equivalent to a free electron gas^36,37^. The ELF analysis indicates that the O–M bonds actually have a low covalency degree, although some covalent bond characteristics did exist between O and its surrounding metallic elements (i.e., the bonds become stiffened), which is in contrast to a full covalent bonding between O and metallic elements in the oxides ZrO_2_ and TiO_2_ (Supplementary Fig. 15). Therefore, oxygen actually acts as an alloying element and makes the O-centered clusters denser through its large atomic radius difference and negative heat of mixing with the constituents, as well as its contribution of a modest degree of covalency through the O-metal bonding.

In the simulations pre-strained to 4%, the atomic shear strain in the base alloy varies in the range from 0.01 to 0.24 (Fig. 5d), indicating an inhomogeneous distribution of local shear strains. In the O-containing alloy, the O-LDPRs (as circled in the inset of Fig. 5e) were subjected to a shear strain 20% lower than the average value, whilst their surrounding regions experience an atomic shear strain up to ten times that of the O-LDPRs. As the macroscopic strain increases to 8%, the distribution curve of the atomic shear strain distribution in the base alloys widens from 0.01 to 0.32 at a standard deviation of 0.037 (Fig. 5d, see Methods section). Surprisingly, the range of atomic shear strain in the O-containing alloy widens from 0.01 to 0.34 at a much larger standard deviation of 0.063 (Fig. 5e), manifesting a larger fraction of atoms having strains different from the average value. This corresponds to a larger degree of strain heterogeneity beneficial for plasticity enhancement of BMGs^38^. The atomic shear strain in the O-LDPRs increases slightly, but dramatically rises in their surrounding regions that accommodated the majority of the plastic strain.

## Discussion

Actually, effects of NMEs, especially oxygen, on GFA and plasticity have been widely studied in a few MG systems^39–47^. Usually, increasing the O content promotes formation of oxides or O-containing precipitates which act as heterogeneous nucleation sites for other intermetallic phases, thus deteriorating the alloys’ GFA and thermal stability^44,45^. Also, formation of such brittle phases and the increase of covalent bonds induce local stress concentrations during deformation, leading to an abrupt drop in plasticity^39–43^. As elaborated above, our new design strategy involves minor addition of small quantities of NMEs. These small elements occupy the energetically preferential interstitial sites while the large negative heat of mixing with BMG constituents enables strong atomic bonding and formation of LDPRs, resulting in a substantial enhancement not only of mechanical performance but also of the GFA. It is important to point out that doping should remain below a critical value to avoid formation of brittle compounds (i.e., oxides, nitrides, borides, and carbides) and to make sure that the NMEs-metal bonds are primarily of metallic nature. Moreover, the critical value of oxygen addition, i.e., the threshold oxygen content, for enhancing ductility in different BMG systems depends on the specific alloy system. In BMG systems containing for example Al, which has strong affinity with O^39–43^, the strong tendency to form Al-oxides would lead to a very low admissible threshold oxygen content, capable for enhancing the GFA and ductility.

**Fig. 6:**
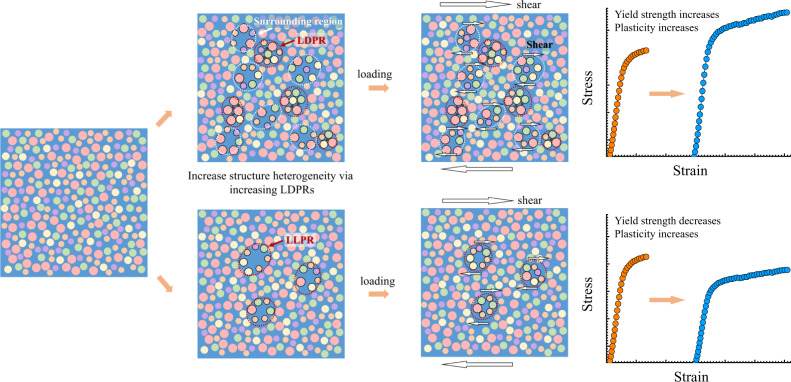
Schematic illustration of two ways to enhance structural heterogeneity of BMGs. One is to increase LLPRs by pre-deformation etc., through which plasticity increases, but at a loss of strength. The other way is to increase LDPRs by doping with NMEs, through which both strength and plasticity are enhanced. Spheres with varied colors and sizes represent different constituents of a multicomponent BMG system.

The deformation behavior is sketched in Fig. 6. Addition of NMEs promotes formation of LDPRs centered around them. These local regions have very low potential energy and behave in a much stiffer manner like “hard spots”. The surrounding regions become relatively loosely packed and have higher potential energy. Consequently, the distribution of the potential energy and atomic packing density in the BMGs was widened and the structural heterogeneity was enhanced. During deformation, the zones surrounding LDPRs are preferentially strained to accommodate the applied stress, and local shear events can subsequently be confined by the hard NME-centered LDPRs. As the deformation proceeds, a higher volume fraction of such local regions gets activated and then increases the plastic flow once they reach the percolation limit^12^. It is important to point out that addition of NMEs increases the density of fertile regions prone to be activated during loading, as clearly demonstrated by the pronounced *γ* relaxation and the increase in plasticity. Nonetheless, the critical stress for activation of percolative plastic flow is increased, leading to the high “pop-in” stress and the higher yield stresses observed.

It is well accepted that the key to enhance the plasticity of BMGs is to increase structure fluctuations in their atomic packing. For traditional methods mentioned earlier, the structure fluctuations is usually enhanced by shifting local atomic packing density downward due to the introduction of more loosely packed regions, as demonstrated by blue dots and line in Supplementary Fig. 16. Obviously, our current approach is to enhance structural fluctuation by introducing LDPRs. The amplitude of fluctuations is dramatically enhanced by shifting local atomic packing density upward at LDPRs (the red line in Supplementary Fig. 16). This effectively creates more possible STZs capable for being activated at high stress. In addition, this strategy is different from the reports about beneficial effects from H addition, in which the slight improvement in plasticity still originated from the formation of more loosely packed regions due to the Troiano’s hydrogen-enhanced decohesion theory^48,49^.

To conclude, the current findings show how the room-temperature brittleness of BMGs can be successfully overcome by a different design concept. This is reached through the formation of plastically compliant regions that form around densely packed clusters containing interstitial dopants. Small interstitial atoms qualify as ‘cluster formers’ for this approach owing to their small size, thermodynamic considerations, and their partially covalent bond contributions. The resulting increase in structural heterogeneity was demonstrated to be an effective way to substantially enhance the plasticity of BMGs, at no loss but rather gain in strength. As a result, appropriate doping with NMEs such as oxygen, boron, carbon, or nitrogen can simultaneously improve plasticity, strength, thermal stability, and even enhance GFA. This combination opens up completely new synthesis, processing and application ranges for plastically compliant and damage tolerant BMGs with a good balance between glass formation, plasticity, strength, and cost.

## Methods

### Materials preparation

The (Zr_20_Cu_20_Hf_20_Ti_20_Ni_20_)_100−*x*_M_*x*_ (M = B, C, N, and O, *x* ≤ 0.5%) master alloys were prepared by arc-melting a mixture of pure metals and metalloids (purity >99.9 wt%), TiO_2_ (99.9 wt%) and TiN (99.9 wt%) in a Ti-gettered high-purity argon atmosphere. Bulk metallic glasses were fabricated by suck casting master alloy ingots to a water-cooled copper mold by arc-melting. The alloy ribbons with a cross section of ~0.02 × 1 mm^2^ were produced by melt spinning of the master alloy ingots using linear wheel surface velocities of 30 m/s.

### Mechanical and thermal properties measurements

Compression tests with the samples of 2:1 in length (3 mm) and diameter (1.5 mm) were conducted in a CMT4305 machine at an engineering strain rate of 0.0002 s^−1^. A small strain gauge was used to calibrate and measure the strain during loading.

Tensile tests of dog-bone shaped cylindrical samples with a gauge dimension of Φ1 mm × 5 mm were carried out on a WDW-200D machine with a maximum load of 200 kN at an engineering strain rate of 0.0002 s^−1^ and a small strain gauge was used to calibrate and measure the strain during loading.

Toughness tests were conducted following the method proposed in literature^50^. Notch samples with a dimension of B (thickness) = 1 mm, W (width) = 2 mm, and S (span) = 8 mm were fabricated for the toughness testes. A straight through notch with a root radius of 100 μm and length of 0.43–0.55 W was made using a diamond wire saw, and then sharpened using a razor-micronotching technique. The fatigue pre-cracking of the samples was conducted on a 3 kN fatigue test machine at the frequency of 20 Hz under a constant load ratio of the minimum to maximum of 0.1 with the stress intensity factor of 20 MPa√m. The length of the notch plus the fatigue pre-crack is close to 0.45–0.55 W after 50,000–100,000 fatigue cycles. Only the samples with one single sharp pre-crack were chosen for the fracture toughness testing, Three-point bending (3PB) tests of the fatigue pre-cracked samples were carried out on a 3 kN MTS ACUMEN3 testing machine at a constant displacement rate of 0.12 mm/min at room temperature. The crack opening displacements (CODs) were monitored across the crack mouth using a clip gage (model of MTS 632.29F-30), mounted between knife edges and affixed to the front of the SENB and fatigue pre-cracked samples.

*The relaxation behaviors* of MGs were characterized by a TA Q800 dynamical mechanical analyzer (DMA). DMA samples have the dimension of 0.05 mm × 1.5 mm × 25 mm, which were cut from the ribbons fabricated by the melt-spinning method. The dynamic modulus E*(*ω*) *=* *E*′(*ω*) + *iE*″(*ω*), where the real part *E*′ and imaginary part *E*″ represent the storage and loss modulus, respectively, was measured by tensile mode with a constant heating rate of 1 K/min and discrete frequency *f* of 1 Hz, 2 Hz, 4 Hz, and 8 Hz in metallic ribbons.

The frequency dependence of the peak temperature for the γ relaxation follows an Arrhenius relation, $f={f}_{0}\;{{\exp}}\;{\left(-\frac{{E}_{{{{\rm{\gamma}}}}}}{{kT}}\right)},$ where *f*_0_ is a constant and *E*_γ_ is the activation energy for the γ relaxation.

The effective activation volume *V*_eff_ can be expressed as ${E}_{\gamma }/{G}_{{{{\rm{\infty }}}}}=1/2{V}_{{{{\rm{eff}}}}}{\gamma }_{{{{\rm{c}}}}}^{2}$, where *G*_∞_ is the unrelaxed shear modulus and *γ*_c_ is the average strain caused by the local relaxation of anelastic process which could be taken as 0.15 as discussed in refs. ^51,52^. Characteristic properties of alloys investigated are listed in Supplementary Table 1.

*Nanoindentation tests* were performed to evaluate heterogeneity in these BMGs using a MTS DCM nanoindentation system equipped with spherical indenters with a radius *R* of 1 μm at a constant strain rate of 0.05 s^−1^ to a depth of 200 nm. No <120 indents were conducted on each sample for statistical analysis of the first pop-in events. The initial stage of the load-displacement curve is elastic and follows the classic Hertzian solution for spherical elastic contacts by $P=\frac{3}{4}{E}_{r}\sqrt{R}{h}^{\frac{3}{2}}$, where *P* is the lord, *E*_*r*_ is the reduced modulus, *R* is the radii of indenter, and *h* is displacement. The reduced modulus is presents as ${E}_{r}={\left[\frac{1-{v}_{s}^{2}}{{E}_{s}}+\frac{1-{\nu }_{i}^{2}}{{E}_{i}}\right]}^{-1}$, with *E*_*i*_ and *v*_*i*_ being the Young’s modulus and Poisson’s ratio for the indenter and *E*_*s*_ and *v*_*s*_ for the specimen. The diamond indenters have elastic constants *E*_*i*_ = 1141 GPa and *v*_*i*_ = 0.07. The HE-MGs has *E*_*s*_ = 95 GPa and *v*_*s*_ = 0.35 for base alloy and *E*_*s*_ = 97 GPa and *v*_*s*_ = 0.35 for O0.2, respectively. The corresponding maximum shear stress occurs roughly at a distance of half the contact radius right under the indenter, given by ${\dot{\tau }}_{{\max }}=0.445{\left(\frac{16{P}_{{{{{\mathrm{pop}}}}}-{in}}{E}_{r}^{2}}{9{\pi }^{3}{R}^{2}}\right)}^{\frac{1}{3}}$.

*Low-temperature heat capacity* *C*_p_ was measured with a Quantum Design physical property measurement system (PPMS), in the temperature range of 2–20 K. The as cast amorphous and crystallized samples, 1.5 mm in diameter with mass of ~20 mg, were carefully polished for good thermal contact and placed on top of a sapphire block of known heat capacity with a thermal grease to ensure good thermal contact. Prior to the sample measurement, we measured the heat capacity of an empty sapphire crystal with the applied grease for a baseline correction. The crystallized samples were obtained by heating the glassy sample to 823 K and held for 30 min for full crystallization.

### Structure characterization

Microstructure and morphology were characterized by a Zeiss Supra-55 field emission scanning electron microscopy equipped with an energy dispersive X-ray spectrometer (EDS). Transmission electronic microscopy (TEM) experiment was carried out with a Tecnai-F30 S-TWIN microscopy. The TEM specimens were first mechanically ground to 50 μm thickness and then twin-jet electro-polished with an electrolyte of 8% perchloric acid + 92% methanol. The amorphous nature was examined by X-ray diffraction using Cu Kα radiation (Rigaku D_max_-RB).

Atom probe tomography and 3D elemental distribution analyses were carried out in CAMECA Instruments LEAP 5000XR local electrode atom probe system. The specimens were analyzed in laser mode, with a specimen temperature of 50 K, a pulse repetition rate of 200 kHz, a pulse energy of 40 pJ, and a detection rate of 0.4% ions per field evaporation pulse. Imago Visualization and Analysis Software (IVAS) version 3.8.0 was used for 3D reconstructions, composition analysis and the creation of iso-concentration surfaces. The sharp tip specimens required for 3D-APT were fabricated by focused ion beam milling on a dual-beam FEI Helios 600.

### Measurement of the contents of added nonmetallic elements

The O and N contents of the studied BMGs were measured with two methods; one is the IGF (inert gas fusion) method by using a LECO Instruments machine with IR detection, and the other is 3D-atom probe tomography (3D-APT), which has an atomic resolution of 1 ppm. The C content was measured by APT and high frequency combustion infrared absorption (HFCIA) methods. The B content was measured by APT and Inductively Coupled Plasma-Atomic Emission Spectrometry (ICP-AES) method.

### Molecular dynamic simulation

To investigate the effect of oxygen atoms on the deformation process, we carried out MD simulations for two amorphous alloys. A Cu_40_Zr_60_ MG containing 13,500 atoms in a cubic box with periodic boundary conditions was equilibrated at 2000 K with an EAM potential in an NPT ensemble^53^, then the sample was cooled to 300 K at a cooling rate of 5 × 10^11^ K/s. A Cu_39_Zr_59_O_2_ sample was also prepared using the same method with the hybrid potential of EAM and comb3^54,55^. We sheared the two samples with the strain rate of 10^8^/s along the direction of yz at 300k, and tracked the deformation processes for analysis.

We define the average atomic number density based on the Voronoi tessellation. For atom *i*, its atomic number density is defined as ${{{{\rm{\rho }}}}}_{{{i}}}=\frac{{{{n}}}_{{{i}}}+1}{\sum {{{V}}}_{{{i}}}}$, where ${{{n}}}_{{{i}}}$ is the sum of Voronoi index, i.e. the coordination number of atom *i*. ${{{n}}}_{{{i}}}+1$ is the number of atoms that makes up a Voronoi cell.$\,{{{V}}}_{{{i}}}$ is the sum of the Voronoi volumes of all the atoms in the ${{i}}$-centered cluster. The average atomic density is ${{{{\rm{\rho }}}}}_{{{{\rm{ave}}}}}=\frac{{\sum }_{{{i}}=1}^{{{N}}}{{{{\rm{\rho }}}}}_{{{i}}}}{{{N}}}$, where ${{N}}$ is total number of the atoms.

The distribution of atomic shear strain was fitted by the Gaussian distribution $p\left(\varepsilon \right)={{{p}}}_{0}+{{{p}}}_{1}{\exp }\left[-\frac{{(\varepsilon -\bar{\varepsilon })}^{2}}{2{\Sigma }^{2}}\right]$, where ${{{p}}}_{0}$ and ${{{p}}}_{1}$ are fitting parameters, $\bar{\varepsilon }$ is the average shear strain, and $\Sigma$ is the standard deviation. A larger $\Sigma$ corresponds to a larger fraction of local sites having strains different from the average value, implying a larger degree of strain heterogeneity.

## Supplementary information

Supplementary Information

## Data Availability

The datasets generated and/or analyzed during the current study are available from the corresponding author on reasonable request.

## Code availability

The code used in this study is based on open source and available from the corresponding author upon a reasonable request.
