# Supplementary material for: Substantially enhanced plasticity of bulk metallic glasses by densifying local atomic packing
Source: Nat Commun. 2021 Nov 12;12:6582. doi: 10.1038/s41467-021-26858-9 (PMC8590062; doi:10.1038/s41467-021-26858-9)
Supplement: Supplementary file 1 — Supplementary Information [file 41467_2021_26858_MOESM1_ESM.pdf]

Supplementary Information for

Substantially enhanced plasticity of bulk metallic glasses by densifying local atomic packing

Yuan Wu[1], Di Cao[1], Yilin Yao[1], Guosheng Zhang[1], Jinyue Wang[1], Leqing Liu[1], Fengshou Li[1], Huiyang Fan[1], Xiongjun Liu[1], Hui Wang[1], Xianzhen Wang[2], Huihui Zhu[1], Suihe Jiang[1], Paraskevas Kontis[3], Dierk Raabe[3], Baptiste Gault[3,4], Zhaoping Lu[1*]

[1]Beijing advanced innovation center for materials genome engineering, State Key Laboratory for Advanced Metals and Materials, University of Science and Technology Beijing, Beijing 100083, China

[2]Institute for Advanced Materials and Technology, University of Science and Technology Beijing, Beijing 100083, China

[3] Max-Planck-Institut für Eisenforschung GmbH, Department of Microstructure Physics and Alloy Design, Max-Planck-Strasse 1, 40237 Düsseldorf, Germany

[4]Department of Materials, Imperial College London, Kensington, London, SW7 2AZ, UK

* Correspondence and requests for materials should be addressed to Z. P. Lu (Email: luzp@ustb.edu.cn)

This PDF file includes:

1. Supplementary Tables 1-3
2. Supplementary Figures 1-16

Supplementary Table 1 Density and hardness of the as-cast alloys with different amounts of nonmetallic dopants. Base alloy reprents $Zr_{20}Cu_{20}Hf_{20}Ti_{20}Ni_{20}$ while Mx stands for the $(Zr_{20}Cu_{20}Hf_{20}Ti_{20}Ni_{20})_{100-x}M_x$ alloy (M=O, B, C and N; x=0.1, 0.2, 0.3, 0.4 and 0.5 at.%)

	Density, g/cm^3	Error bar	Hardness, Hv	Error bar
Base alloy	8.79	0.032	484.0	10.10
O0.1	8.82	0.045	508.8	8.00
O0.2	8.85	0.043	521.1	7.10
O0.3	8.83	0.020	519.0	4.47
O0.4	8.79	0.052	514.3	2.65
O0.5	8.78	0.018	510.3	3.38
B0.1	8.83	0.010	487.0	5.11
B0.2	8.90	0.038	492.4	8.08
B0.3	8.86	0.161	503.5	5.84
B0.5	8.70	0.051	500.6	4.74
C0.1	8.82	0.081	501.7	4.10
C0.2	8.85	0.085	509.6	3.67
C0.3	8.86	0.024	510.0	3.26
C0.5	8.75	0.109	511.2	8.35
N0.1	8.85	0.080	508.7	3.04
N0.2	8.78	0.001	514.9	2.52
N0.3	8.78	0.012	511.7	3.05
N0.4	8.75	0.078	509.2	3.87
N0.5	8.76	0.052	510.0	3.98

Supplementary Table 2 Measured values of nonmetallic elements for the alloys studied.

	From APT, at. %	From IGF (O,N), HFCIA (C), ICP-AES (B), at. %
Base alloy	0.081%	0.049%
O0.1	0.172%	0.168%
O0.2	0.265%	0.279%
O0.3	0.359%	0.392%
O0.4	0.472%	0.483%
O0.5	0.585%	0.571%
B0.1	0.112%	0.130%
B0.2	0.223%	0.195%
B0.3	0.307%	0.389%
C0.1	0.086%	0.168%
C0.2	0.174%	0.221%
C0.3	0.281%	0.292%
N0.1	0.151%	0.142%
N0.2	0.233%	0.282%
N0.3	0.343%	0.326%

Supplementary Table 3 Characteristic properties of alloys investigated, including unrelaxed shear modulus, G_{∞}, activation energy $E_{\beta'}$ for the β' relaxation, the effective volume V_{eff} obtained with $\gamma_c = 0.15$.

	$E_{\beta'}$ (eV)	G (GPa)	$E_{\beta'}/G = 0.5\ \gamma_c^2 V_{\mathrm{eff}}$ (Å^3)	V_{eff} (nm^3)
base alloy*	0.91±0.08	36.5	3.96	0.35
O0.1	0.65±0.11	37.0	2.80	0.25
O0.2	0.64±0.08	37.2	2.59	0.23
O0.3	0.76±0.07	37.2	3.29	0.29
B0.2	0.59±0.05	37.3	2.53	0.23
N0.2	0.68±0.09	36.9	2.79	0.25
C0.2	0.69±0.09	37.0	2.99	0.27

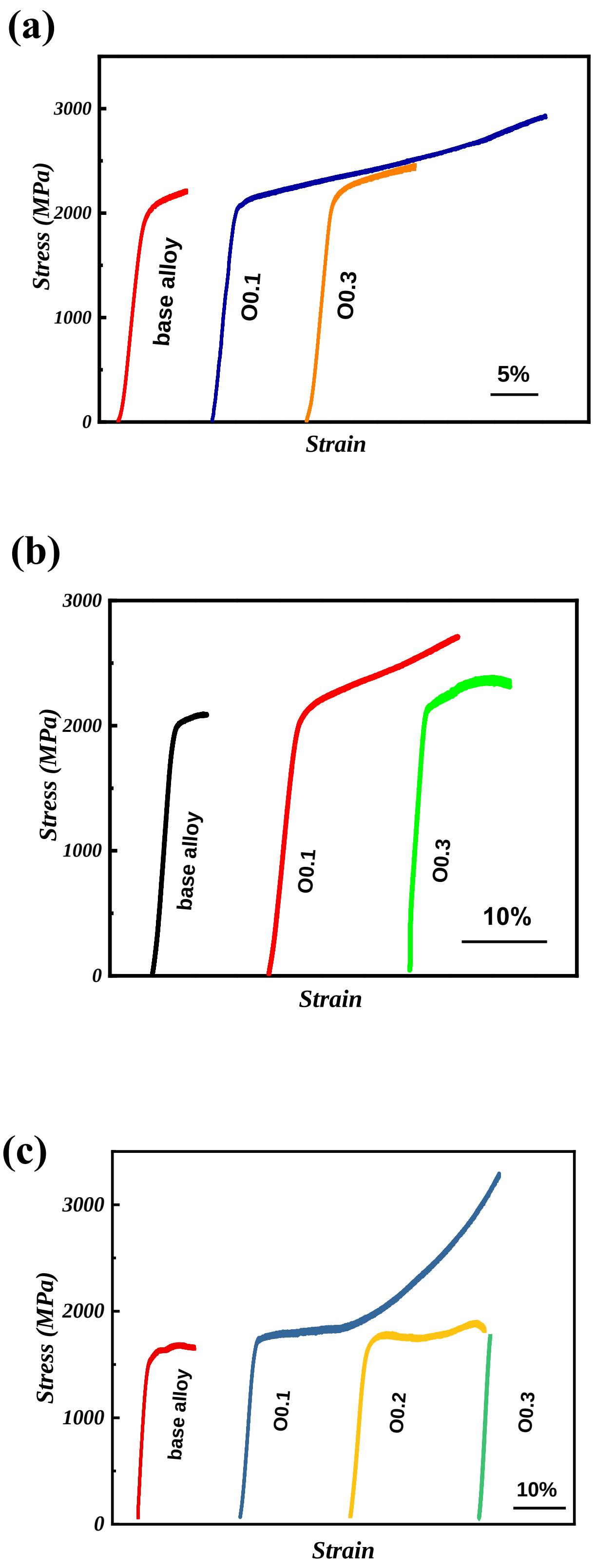

Supplementary Figure 1 Compressive stress-strain curves of $Ti_{42.5}Cu_{40}Zr_{10}Ni_5Sn_{2.5}$ (a), $Cu_{42.34}Ti_{32.38}Zr_{7.6}Ni_{9.28}Hf_{8.4}$ (b) and $Zr_{33.2}Cu_{16.7}Ti_{16.7}Ni_{16.7}Hf_{16.7}$ (c) with a different content of oxygen.

(a)

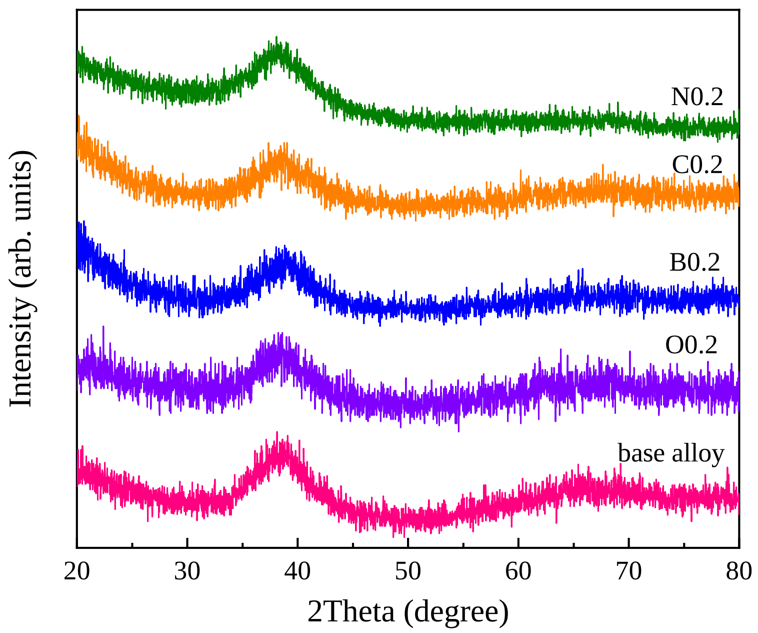

(b)

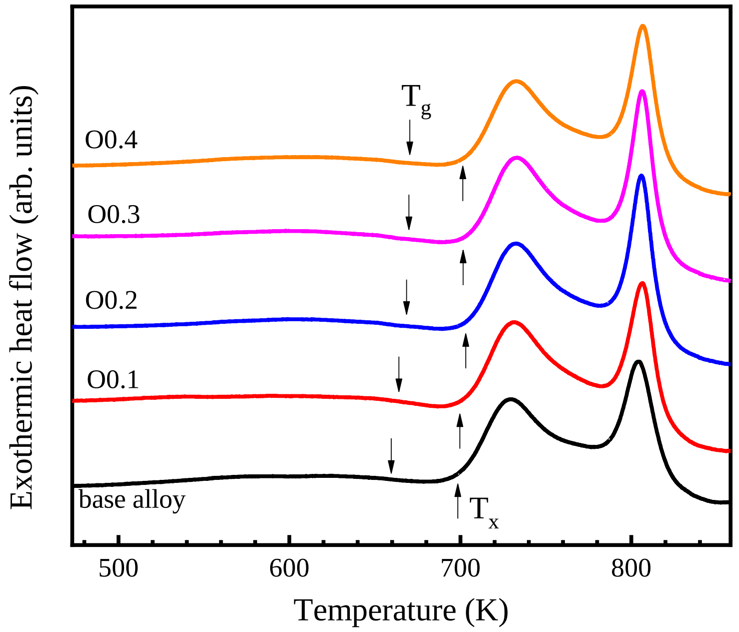

Supplementary Figure 2 (a) XRD spectra of the as-cast $Zr_{20}Ti_{20}Hf_{20}Cu_{20}Ni_{20}$ alloy and the variants containing 0.2 at.% of different alloying additions. All samples show only diffuse humps, indicating a fully amorphous structure; (b) DSC curves of base alloy and O-doped alloys.

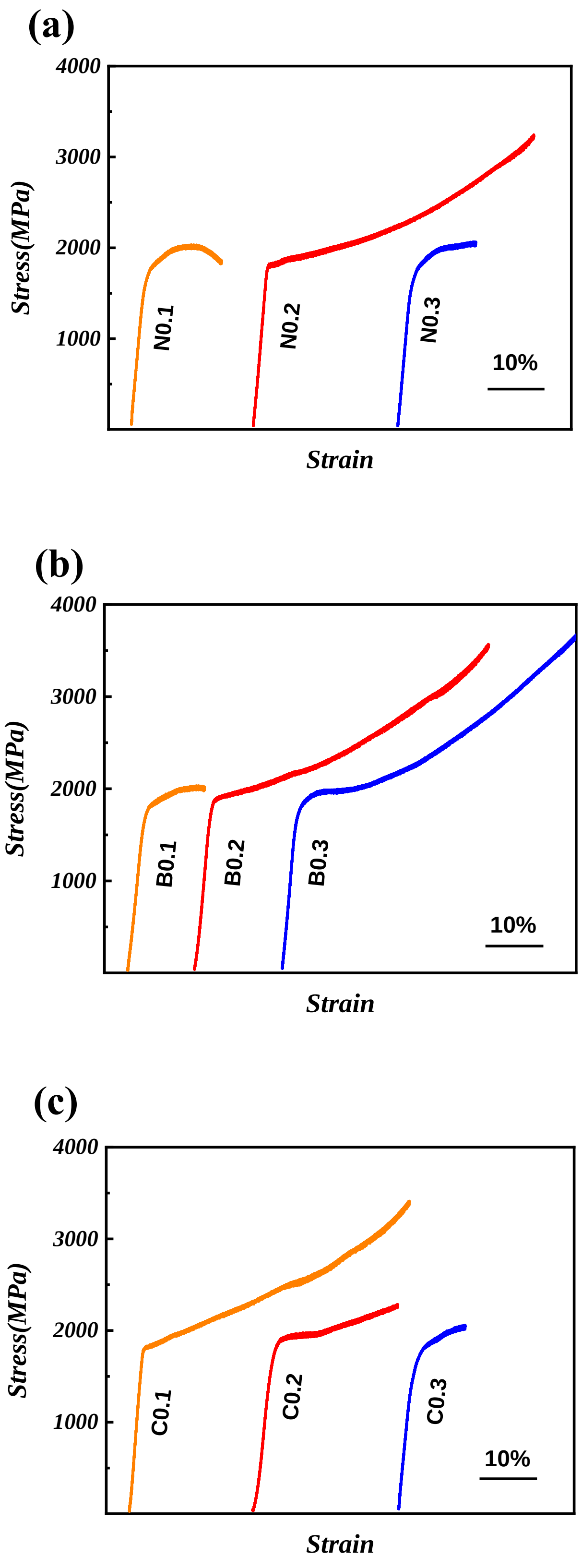

Supplementary Figure 3 Stress-strain curves of the ZrTiHfCuNi BMGs doped with different contents of N (a), B (b), and C (c). Doping of the BMGs with 0.1-0.2 at.% of

these small interstitial atoms was found to dramatically enhance the plasticity of the resultant BMGs.

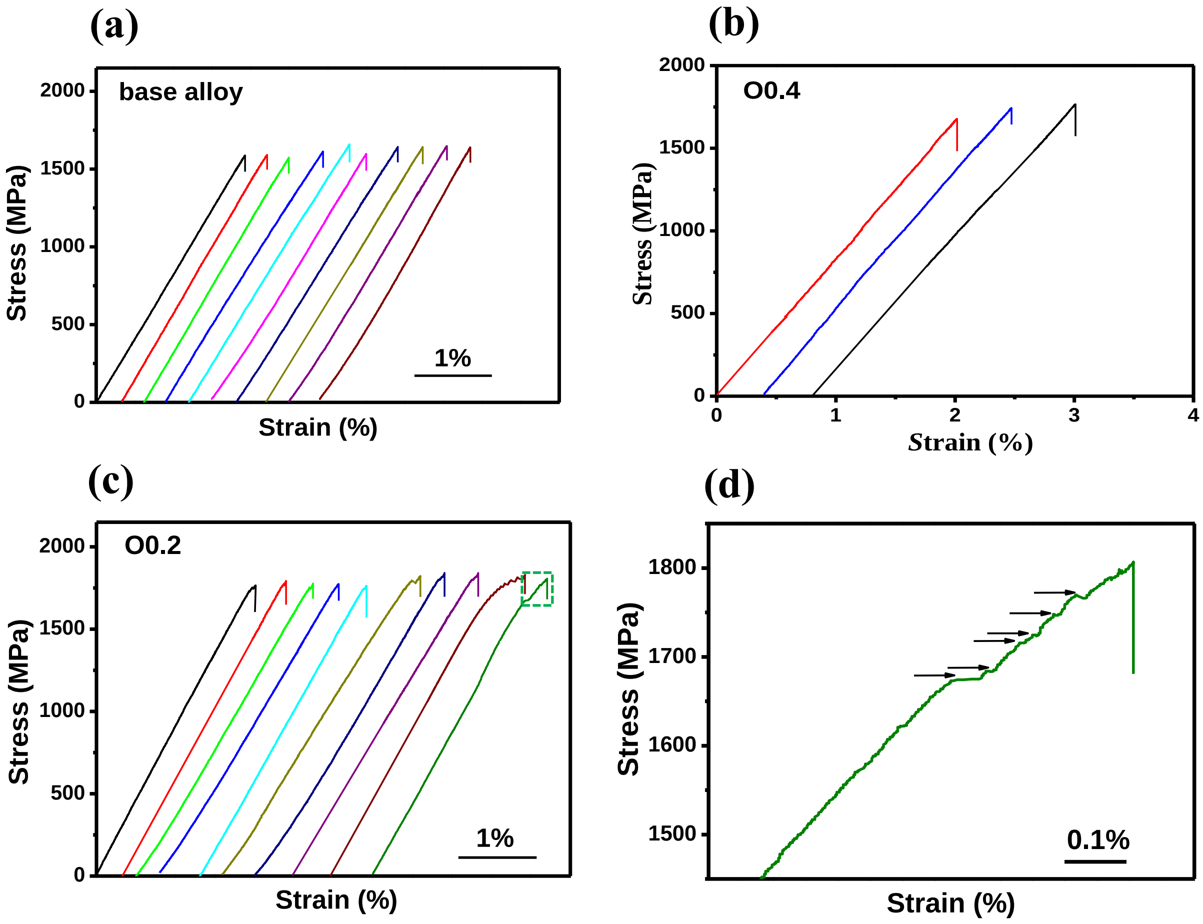

Supplementary Figure 4 Tensile stress-strain curves of the as-cast base alloy (a), O0.4 (b), O0.2 (c) and the magnified view (d) of the tensile stress-strain curve in the vicinity of yielding of a representative sample in (c).

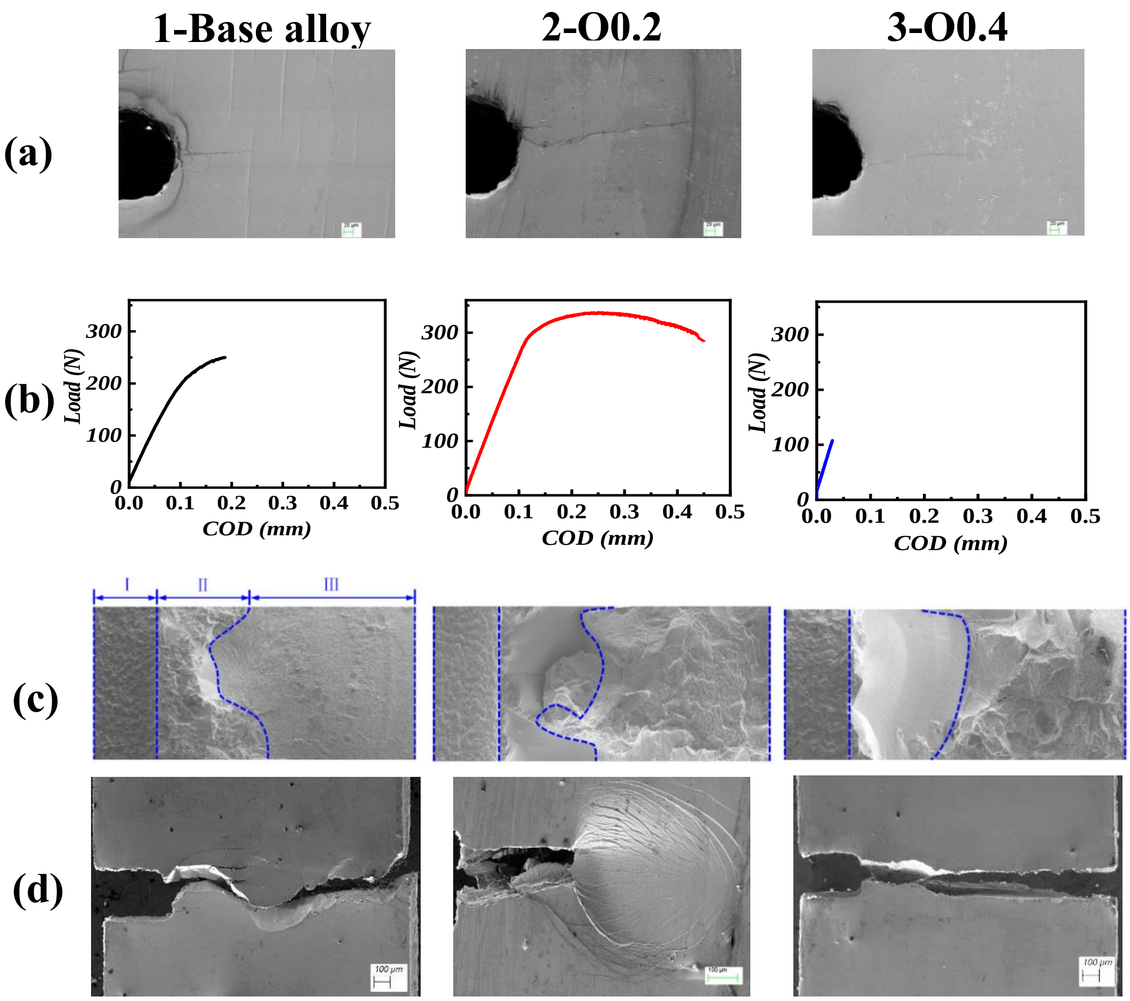

Supplementary Figure 5 (a) figure pre-crack of the base alloy, O0.2, and O0.4, (b) 3PB load-COD curves of base alloy O0.2, and O0.4, (c) SEM images of the fracture surface after 3PB tests of the base alloy, O0.2 and O0.4, respectively, showing the pre-notched (I), fatigue pre-cracked (II) and overloaded (III) regions, and (d) the area in front of the crack after 3PB of the base alloy, O0.2, and O0.4, respectively

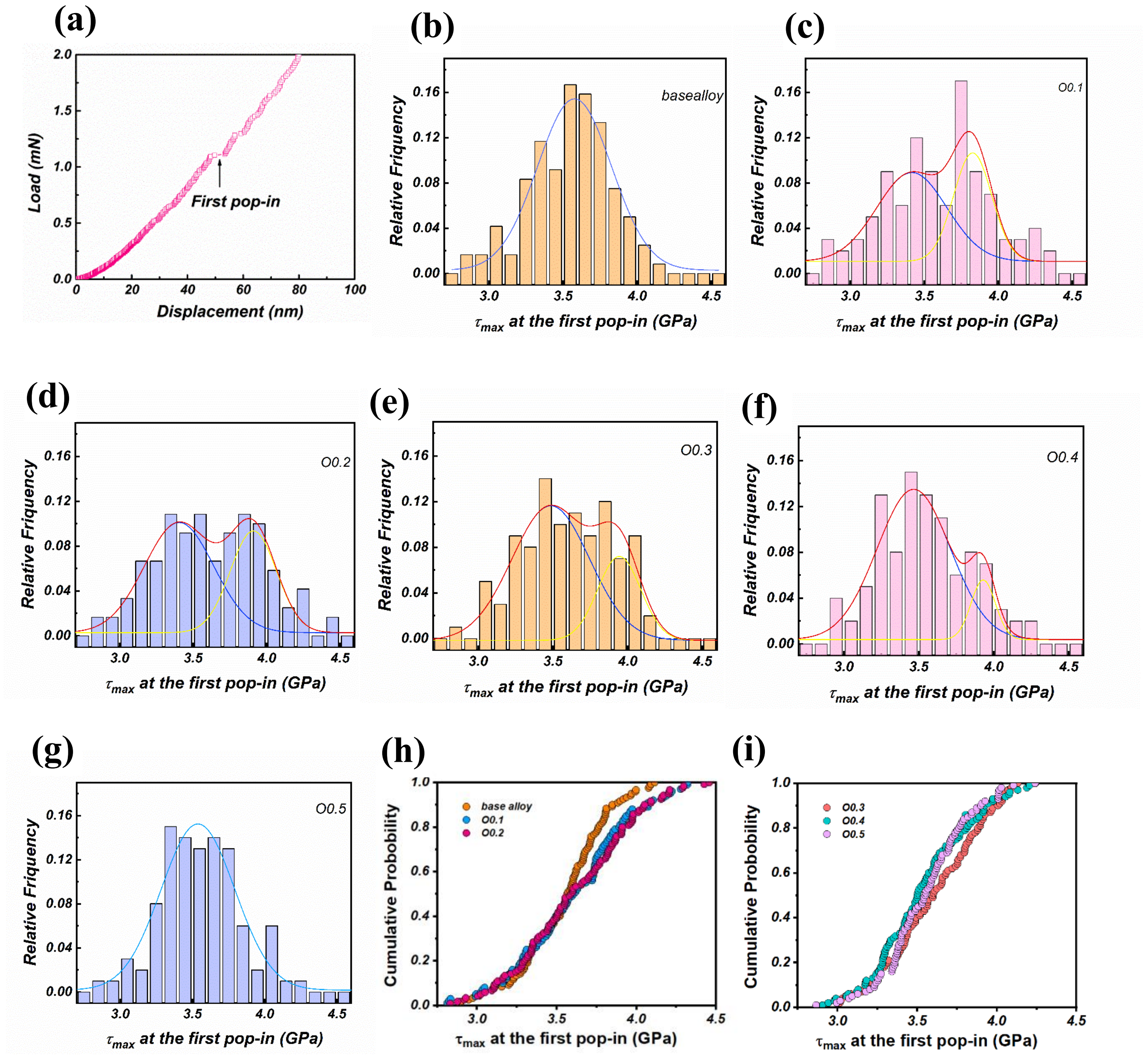

Supplementary Figure 6 A representative load-displacement curve obtained from nano-indentation, and the first pop-in signifies the onset of the plastic deformation during indentation (a). Relative frequency distributions of τ_{max} in nanoindentation of base alloy (b), O0.1 (c), O0.2 (d), O0.3 (e), O0.4 (f) and O0.5 (g), and the lines represent the Gaussian fitting of measured data. (h) and (i) show the corresponding cumulative probability of τ_{max} in (b-g) respectively.

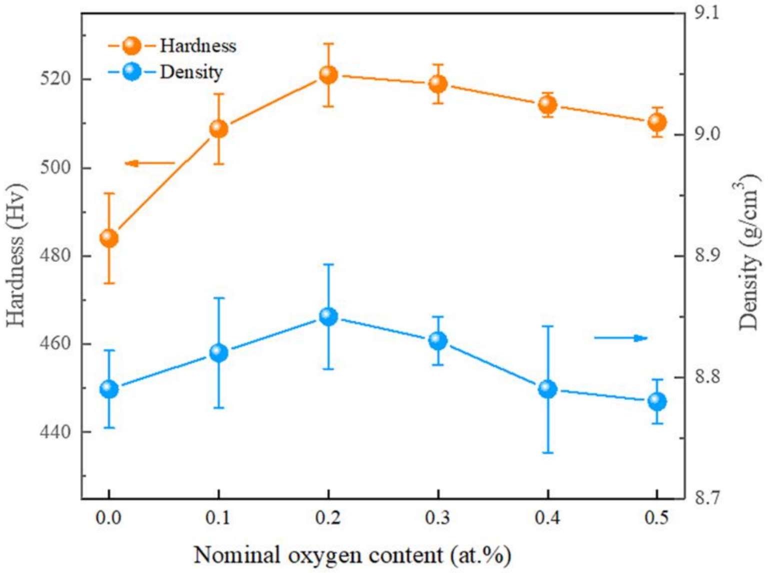

Supplementary Figure 7 Variation of hardness and density with the content of oxygen in $Zr_{20}Cu_{20}Hf_{20}Ti_{20}Ni_{20}$ BMG and its variants. The error bars were estimated from standard deviation with a confidence of 95%

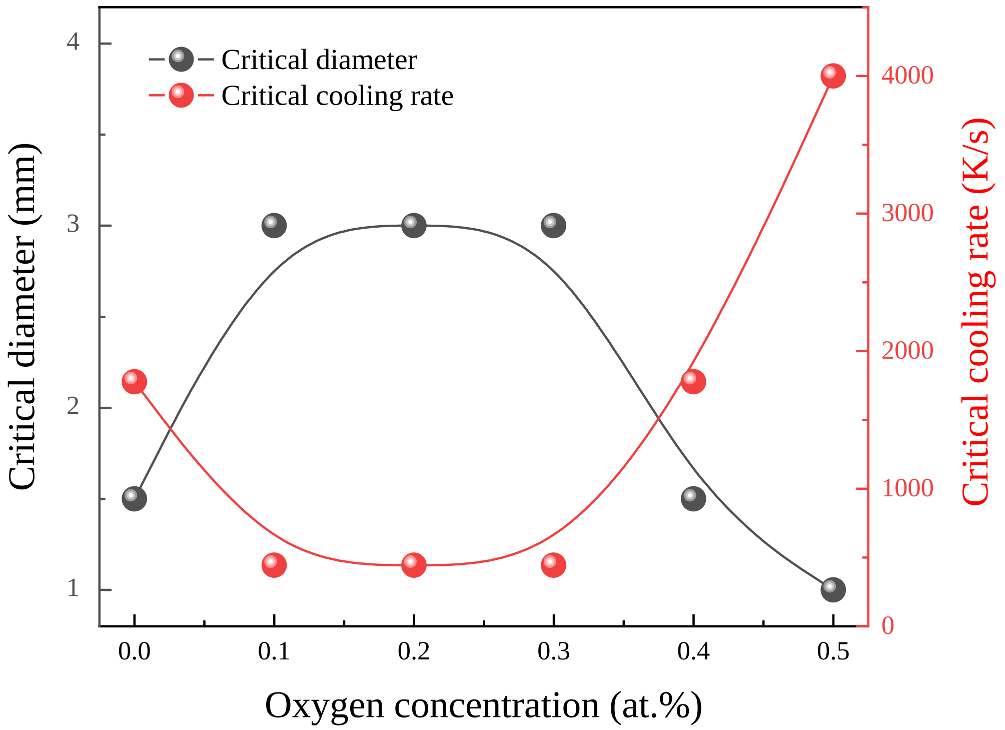

Supplementary Figure 8 The attainable maximum diameter (D_{max}) for glass formation and the critical cooling rate (R_c) of $Zr_{20}Cu_{20}Hf_{20}Ti_{20}Ni_{20}$ BMGs with different amounts of oxygen. The critical cooling rate was estimated by $R_c=10/R_{max}^2$ (R_{max} represents the attainable maximum radius in centimeter for a given alloy) proposed by Lin et. al[1, 2].

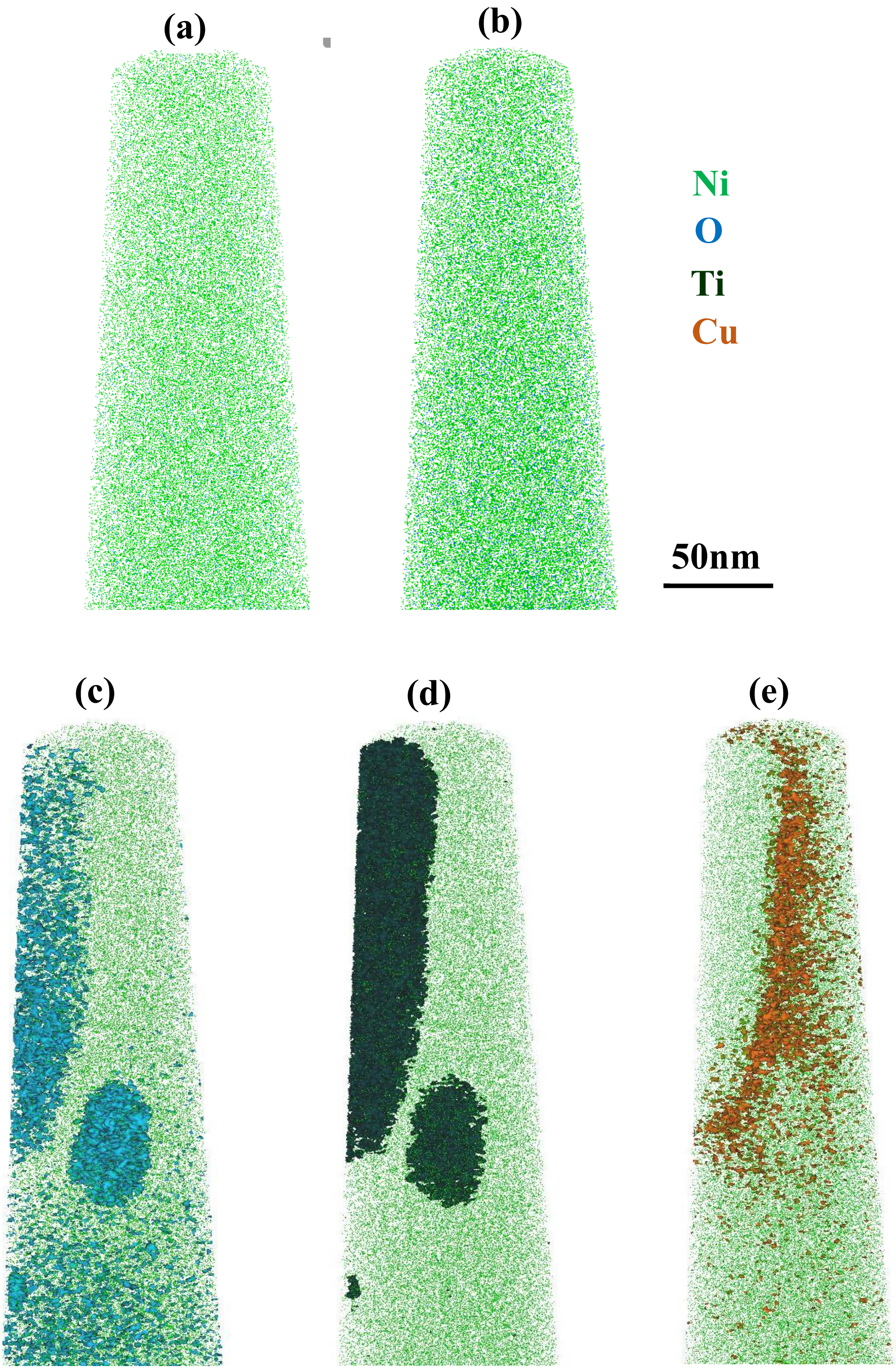

Supplementary Figure 9 3D-APT images of base alloy (a) and O0.2 (b). Only Ni and O atoms were illustrated for simplification. Elemental iso-surfaces of O0.5 with 1.8% O (in light blue) (c), 26% Ti (in dark green) (d) and 22.5% Cu (in orange) (e) are also shown.

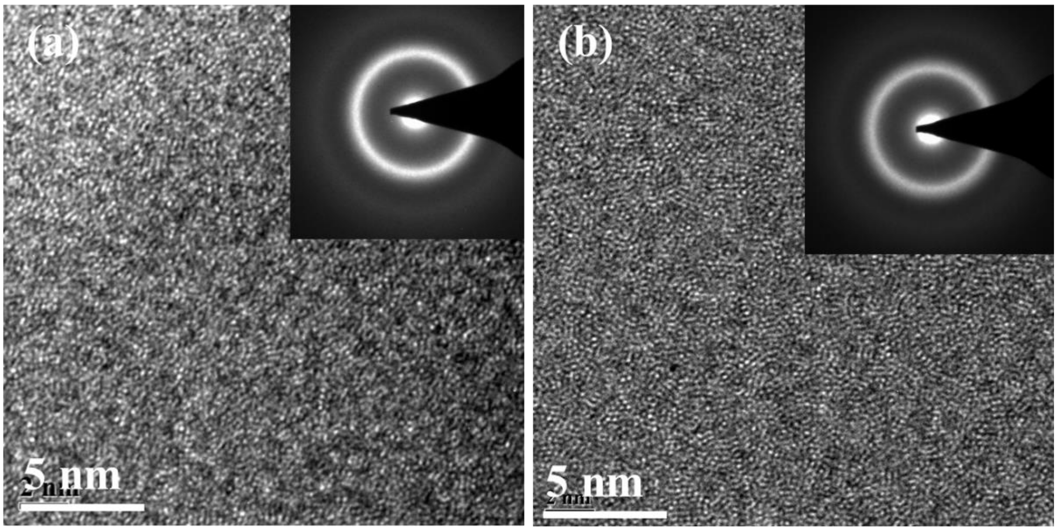

Supplementary Figure 10 HRTEM and SEAD images for the as-cast, 1.5 mm rods of (a) base alloy and (b) O0.2.

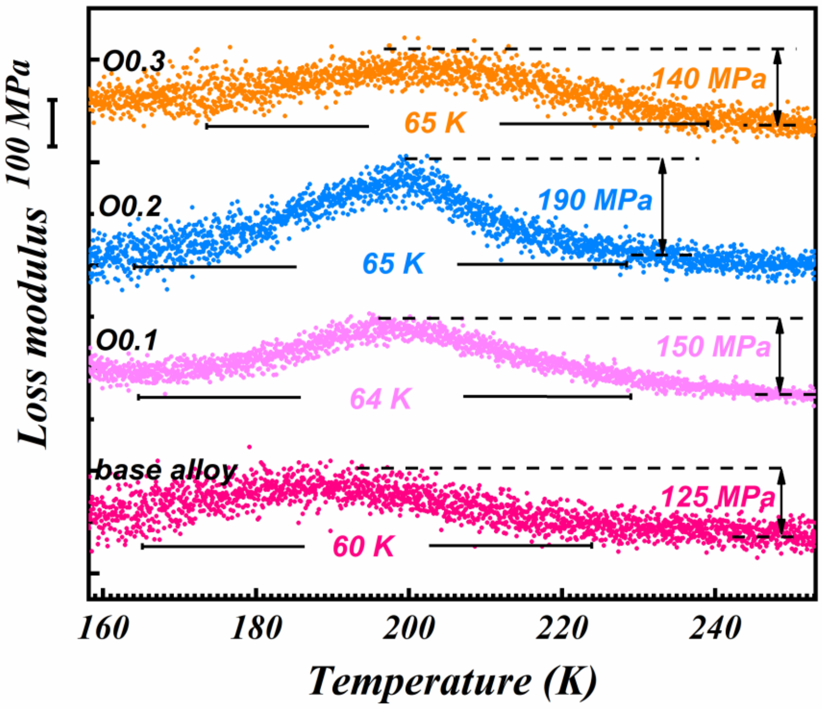

Supplementary Figure 11 Determination of the height (*H*) and the width (*W*) of the fast *β*' relaxation peaks on the loss modulus *E''*-*T* curves obtained at a constant heating rate of 1 K/min and a constant frequency of 1 Hz for the base alloy and O-doped alloys.

(a)

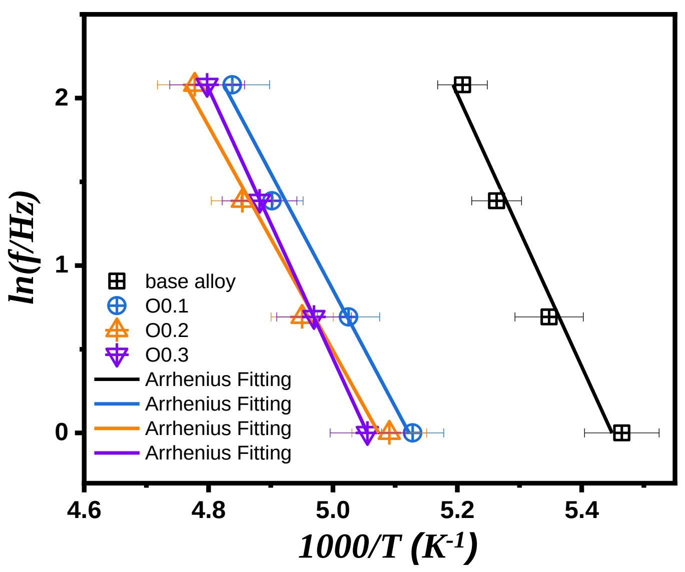

(b)

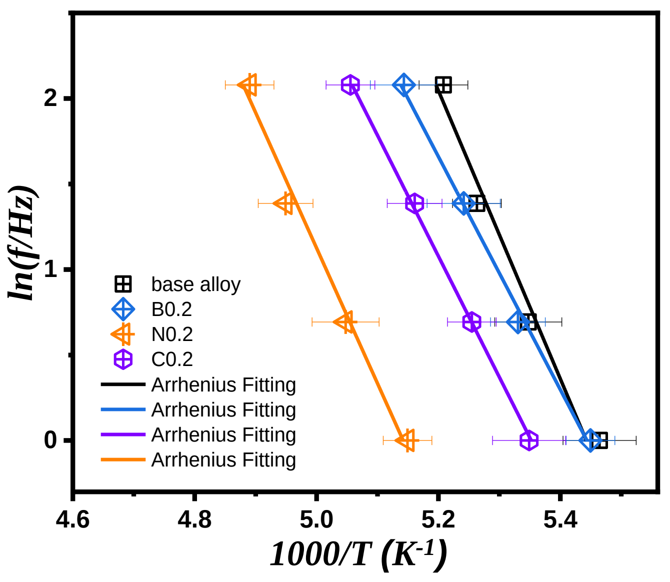

Supplementary Figure 12 Plots of frequency dependence of the peak temperature of the β' relaxation via the Arrhenius relation. Activation energy of the β' relaxation can be obtained from the plots for ZrTiHfCuNi BMGs doped with different contents of small atoms of (a) O, (b) B, N, and C. The error bars for the peak temperature were estimated from standard deviation with a confidence of 95%.

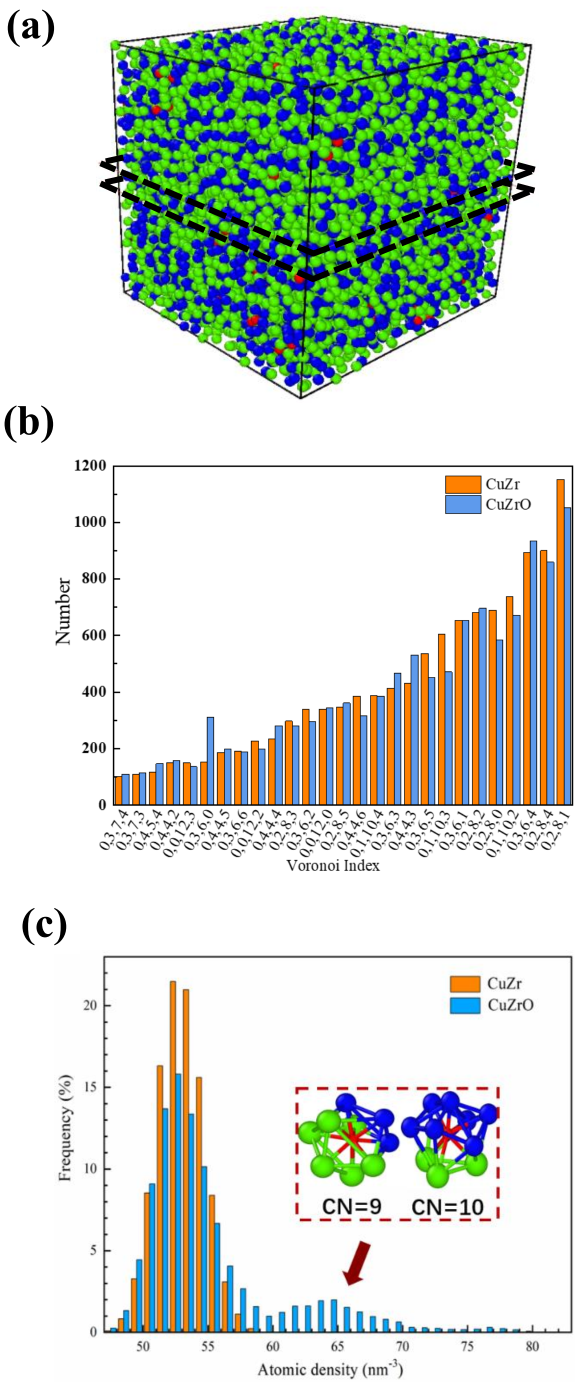

Supplementary Figure 13 The three dimensional atomic configuration with 13500 atoms, and the location where the slice in Fig. 3 was taken from (a). Number of polyhedra in the base $Zr_{60}Cu_{40}$ alloy and the alloy with O addition (b). After doping of O, the number of clusters with Voronoi index of (0,3,6,0) dramatically increases. Distribution of atomic number density in the base and O-doped alloy (c), and the inset shows two representative O-centered clusters. O-centered regions are efficiently packed and their average atomic number density ranges from 65 to 82 nm^{-3}, whilst most of their surrounding regions have a much lower atomic number density ranging from 46 to 65

nm^{-3}. A majority of LDPRs around O were found to have a coordination number (CN) of 9 and 10 with a Voronoi index of (0,3,6,0) and (1,3,3,3), respectively.

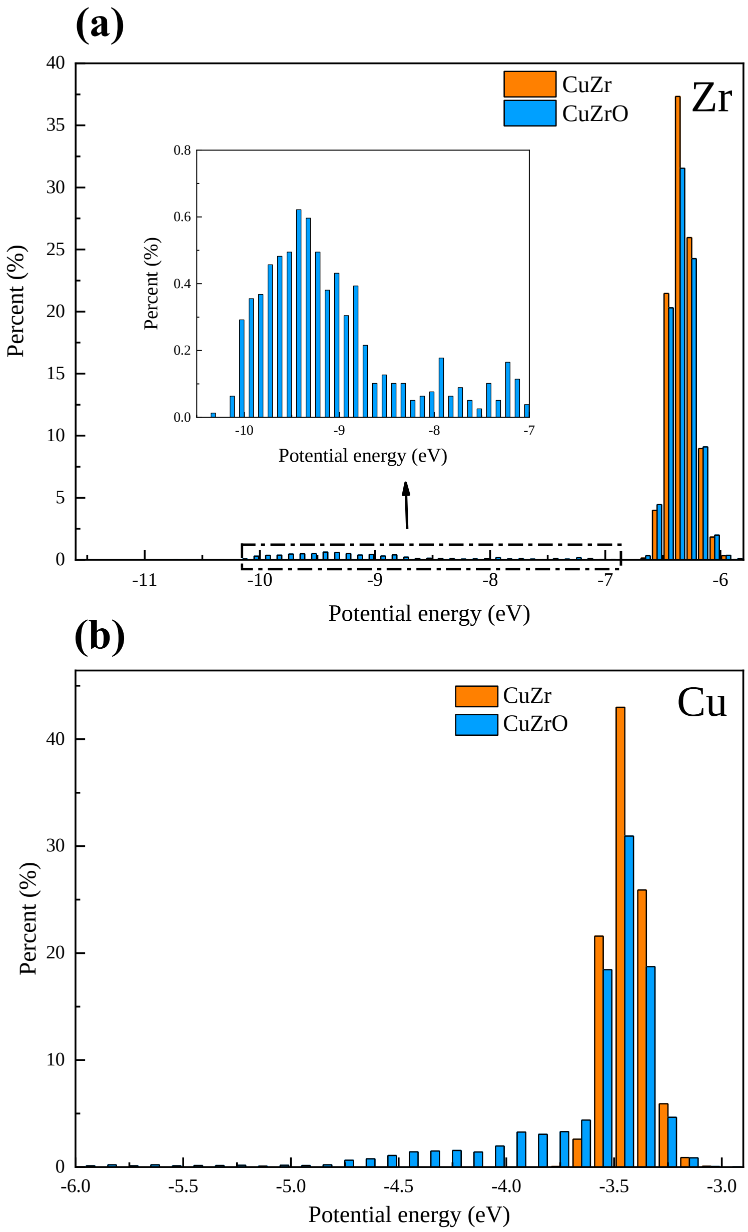

Supplementary Figure 14 Distribution of the atomic potential energy of Zr (a) and Cu (b) atoms in the base and the O-containing alloy. The potential energy of Zr and Cu atoms in the oxygen-containing alloy has not only a much wider distribution, but also an extra small hump at much lower energy which corresponds to the Zr and Cu atoms around O.

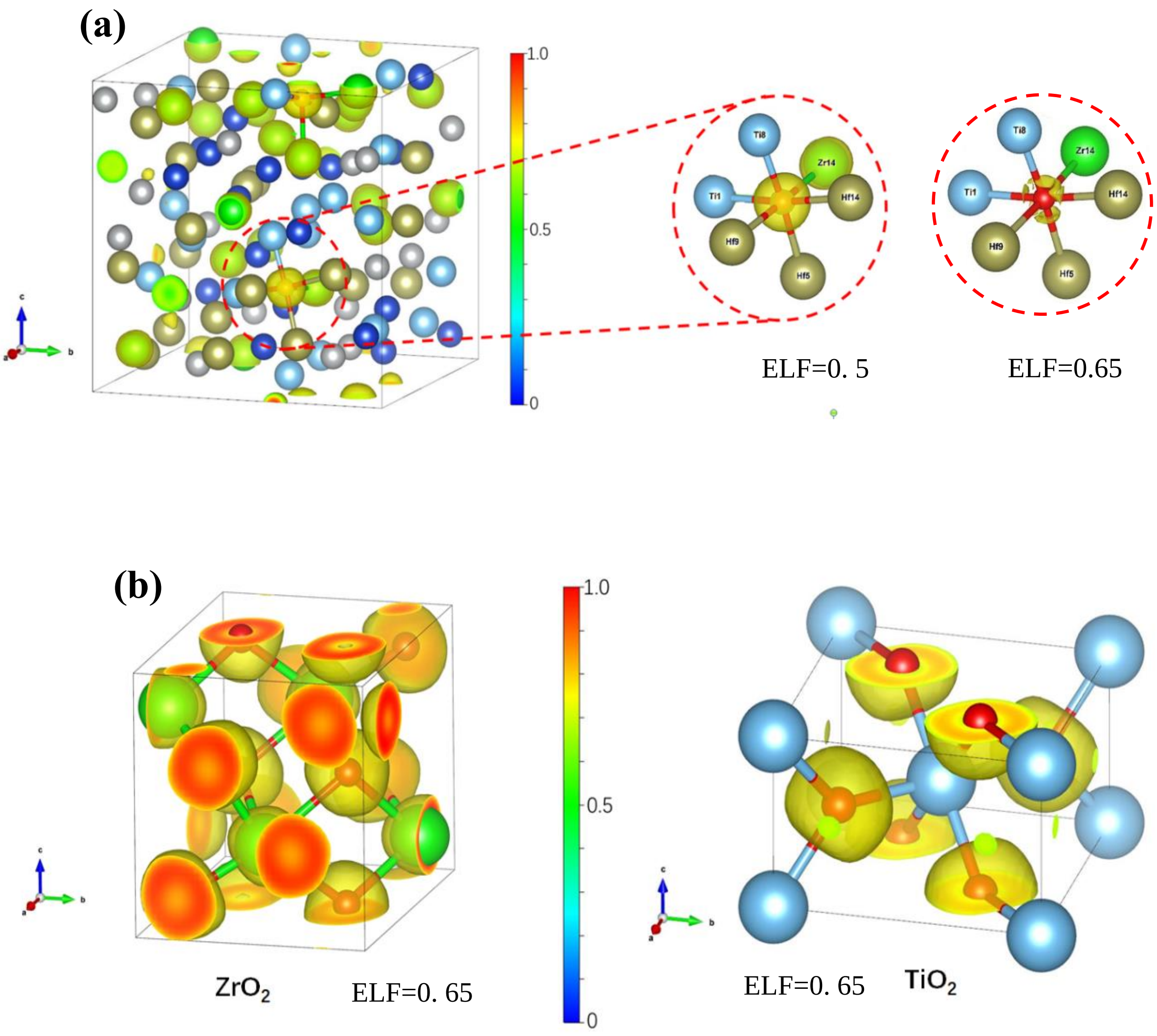

Supplementary Figure 15 ELF (electron localization function) calculation of O-doped TiZrHfCuNi with isosurface values of 0.5 and 0.65 (a), and TiO2 and ZrO2 with an isosurface value of 0.65 (b).

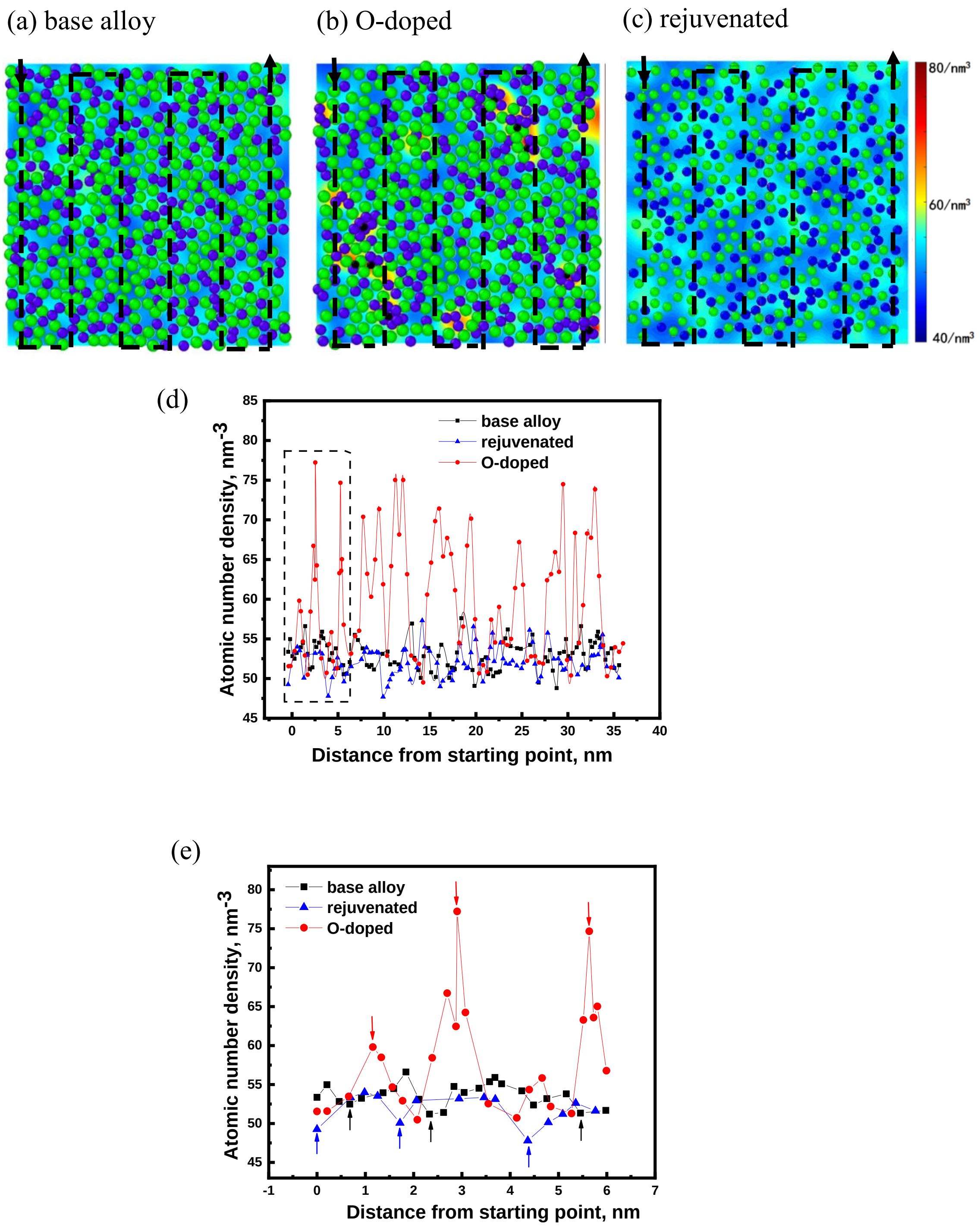

Supplementary Figure 16 Atomic number density of the un-doped base reference alloy, O-doped alloy and rejuvenated alloy. Slices (6.25×6.25x×0.35 nm3) taken from MD simulation as a representative example of atomic packing in un-doped base alloy (a), O-doped alloy (b) and rejuvenated alloy(c). (d) Line profiles of local atomic packing density taken along the dashed lines from respective samples in (a), (b) and (c). (e), one representative enlarged segment in the circled regions in (d) for a more detailed

illustration. The arrows in (e) indicate the local maxim (red) or minim (black and blue) in the profile of local atomic packing density.

Supplementary References

1. Cao, D., Wu, Y., Li, H. X., Liu, X. J., Wang, H., Wang, X. Z., Lu, Z. P., Beneficial effects of oxygen addition on glass formation in a high-entropy bulk metallic glass. Intermetallics 99, 44-50 (2018).
2. Lin, X.H., Johnson, W.L., Formation of Ti-Zr-Cu-Ni bulk metallic glasses. J. Appl. Phys., 78, 6514-6519 (1995).
